# The Impact of Artificial Intelligence on the Mental Health of Manufacturing Workers: The Mediating Role of Overtime Work and the Work Environment

**DOI:** 10.3389/fpubh.2022.862407

**Published:** 2022-04-11

**Authors:** WanQing Wei, LinYu Li

**Affiliations:** School of Public Administration, Guangxi University, Nanning, China; Regional Social Governance Innovation Research Center, Guangxi University, Nanning, China

**Keywords:** artificial intelligence, mental health, overtime work, work environment, manufacturing industry

## Abstract

**Background:**

Work-related mental health and psychological injuries are important issues related to people's livelihood and wellbeing. Currently, digitalization and intelligent technology have an extremely large impact on the workforce. China is actively promoting the deep integration of artificial intelligence (AI) and manufacturing, which may have important implications for the mental health of manufacturing workers. However, existing researches have paid little attention to the influence of AI on the mental wellbeing of workers in China. There is a lack of relevant empirical research, and the findings in existing studies are inconsistent.

**Methods:**

Using data from the 2018 China Labor Force Dynamics Survey, this paper studies the impact of AI on the depressive symptoms of manufacturing workers and uses stepwise and bootstrapping methods to test whether overtime work and the work environment exhibit mediating effects. Robustness tests were performed by using alternative measures for the dependent and mediating variables. Finally, the heterogeneity in the impact of AI by skill level and generation was examined.

**Results:**

AI can reduce the psychological depression scores of manufacturing workers by 1.643 points, which indicates that AI promotes workers' mental health. Working overtime is not a mediator between AI and mental health. However, the work environment is a mediator between AI and the mental health of manufacturing workers: it explains 11.509% of workers' mental health. The impact of AI on the mental health of manufacturing workers varies by skill level and generation. AI improves the mental health of low-skilled manufacturing workers by 2.342 points and that of manufacturing workers born before the 1980's by 2.070 points.

**Conclusions:**

The application of AI is conducive to improvements in the mental health of manufacturing workers. Improving the work environment is a powerful way to increase the positive effects of AI on workers' mental health. The impact of AI on the mental health of manufacturing workers varies by skill level and generation. The mental health of low-skilled workers and workers born after 1980 is affected more positively by the adoption of AI.

## Introduction

Since 2015, the Chinese government has promoted a master plan entitled “Made in China 2025,” which emphasizes the development of “intelligent manufacturing” through the utilization of AI (1, 2). China's AI industry reached 303.1 billion yuan in 2020, up 15% year-on-year, which indicates growth that is slightly faster than the global growth rate. In the Chinese context, the rapid adoption of AI in manufacturing is bound to have profound impacts on working conditions, labor relations, and the mental health of employees (3). Therefore, it is highly important that the ways in which the adoption of AI affects Chinese manufacturing employees' mental health is explored. However, there are few empirical studies on this topic. Moreover, although some researchers have begun to study the impact of AI on workers, their findings are inconsistent and even conflicting. On the one hand, some researchers view the utilization of AI as an important way to alleviate the “contradiction between family and work” and to achieve “balance between family and work” (4, 5). Therefore, they are optimistic about the impact of AI on workers' mental health. On the other hand, other researchers have argued that technological upgrading is being used as a strategy to substitute capital (i.e., technology) for labor in the face of increasing labor bargaining power and labor costs (6). AI helps reduce dependence on workers by replacing jobs and reducing the need for human labor. Moreover, “deskilling” through AI reduces the bargaining power of workers (7). Thus, AI changes the balance of power in labor-capital relations through employment substitution and deskilling, which further increases the pressure on workers to remain employed and negatively impacts their mental health (8).

Given the literature reviewed above, this paper explores the impact of AI on the mental health of employees in the Chinese manufacturing industry. Specifically, we focus on the following questions. First, how does the adoption of AI in manufacturing affect the mental health of employees? At this stage, is the use of AI beneficial or detrimental to the mental health of employees? Second, if the utilization of AI has a significant effect on the mental health of employees, what is the specific mechanism or pathway by which this effect occurs? Furthermore, is this impact balanced across workers with different skill levels and in different generations? After combing the literature that explains the effects of AI on employment and on mental health, we argue that in its current stage, intelligent development in the manufacturing industry is mainly used as a substitute for some highly labor-intensive jobs and jobs with unfavorable working environments. Such substitution helps increase work efficiency and improve work environments in the manufacturing industry. Simultaneously, given the labor shortage in the manufacturing industry, the impact of employment pressure on workers' psychological health is extremely limited. This paper uses nationally representative data from the *China Labor Force Dynamics Survey* (CLDS) to answer the above questions.

The possible contributions of this paper are as follows. First, against the background of labor shortages and rising labor costs, AI adoption become important for the Chinese manufacturing industry. Given this context, the ways in which AI affects workers' psychological health need scholarly attention. This is an issue that must be addressed through government management, policy research and adjustments to labor relations in the current digital and intelligent technology era. However, relevant research is still lagging behind. This paper focuses on the impact of AI on workers' mental health, which is a forward-looking concern. Second, this paper emphasizes the key roles of overtime work and the work environment in mediating the effect of AI on mental health, which contribute to our understanding of the mechanisms underlying AI's impact on mental health. Third, this paper focuses on the heterogeneity in the impact of AI on mental health and finds that this impact varies according by skill level and generation.

The remainder of this paper is organized as follows. In Literature Review and Research Hypotheses, we review the literature that focuses on the impact and mechanism of AI on the mental health of manufacturing workers and propose our research hypotheses. Data, Variables and Models describe our data source, data processing, variables and model setting, as well as our analytical strategy. Results analyse the empirical results, focusing on results related to our hypotheses about the relationships between AI and workers' mental health, the roles of overtime work and work environment in AI on mental health of manufacturing workers.

In Further Discussion: Heterogeneity Analysis, we further explore the heterogeneity in the impact of AI on the mental health of manufacturing workers in terms of skill differentiation and generational differences. Finally, we draw our conclusions.

## Literature Review and Research Hypotheses

### The Impact of AI on Manufacturing Workers' Mental Health

Digitalization and intelligent technology, as the key forces driving the change in the relationship between production and life, have garnered attention and aroused debate in academic circles. Due to rising labor costs and labor shortages, intelligent technology has become widespread in the manufacturing industry, and the deep integration of intelligent technology and manufacturing has become commonplace. However, the academic community has lacked acumen in its attempts to answer the question of how this trend impacts workers' mental health, and conclusions are inconsistent.

Some scholars have explained the impact of AI–manufacturing integration on workers' mental health from the perspective of labor-capital relations and have drawn pessimistic conclusions. They have argued that the intelligentization of manufacturing will reduce workers' employment opportunities and wage bargaining power through technical unemployment and deskilling, which will negatively impact workers' mental health. In the context of labor shortages, intelligent development is a strategy by which capital in the form of technology can be used as a substitute for labor to weaken laborers' bargaining power and reduce labor costs. AI is expected to accelerate innovation and productivity growth. Under the assumption of rationality, enterprises can be expected to increase their use of AI and reduce their labor demand (9). Unlike the limited substitution of specific work tasks during the previous industrial and digital revolutions, AI aims to replace, supplement and/or amplify almost all tasks performed by humans. Therefore, the impact of AI on employment is expected to be stronger than that of the previous technological revolutions (10). AI is not only expected to replace jobs in the labor market but also to change the ways in which all occupations complete their tasks, leading to massive technology-driven unemployment in the future (11, 12). Some scholars have found that empirically, industrial robots have had a greater impact than capital and other technological advances on the U.S. labor market, and the use of industrial robots has had a stronger negative impact on the employment-to-population ratio and the wage level in some U.S. industries (13). In the next 10 or 20 years, approximately 47% of the U.S. workforce is projected to be at high risk for computerization, a figure that includes not only workers in the transportation, logistics and production industries but also most office and administrative support workers (14). In addition, with the rapid development of AI, experts predict that in the next 30 years, AI will transition into super AI, which has one-third probability of negatively influencing humans (15). With AI approaching or reaching human levels of intelligence, it competes fiercely with workers for job opportunities, aggravating the uncertainty in the labor market. Workers are constantly worried about losing their jobs, seeing their incomes fall, and being exposed to economic insecurity. These factors threaten workers' mental health (16). Moreover, AI is expected to impact the traditional career paths of workers, and workers are more likely to respond with resignation, cynicism and depression (17). In addition, due to the replacement of workers with AI, unemployed individuals may experience prolonged unemployment spells and may even fall into permanent unemployment, which will further hinder their ability to meet their social and psychological needs and lead to the deterioration of their mental health.

Some scholars have criticized the above views, arguing that existing research overestimates the potentially destructive power of AI over employment. In contrast, they argue, AI enhances workers' positive emotions by creating jobs, improving efficiency, and increasing incomes. The potential destruction of employment due to AI can also benefit humans, and these benefits will improve mental health. Given that in most jobs, it is difficult for AI to quickly acquire the large amount of tacit knowledge that is needed, the impact of AI on employment has been limited, and only a few occupations are likely to become fully automated in the short or medium term (18). The relatively low average rate of job automation in 21 OECD countries also provides empirical proof for this (19). The jobs that AI replaces or eliminates often involve specific tasks. In other words, not all human jobs can be replaced (20). These jobs usually involve dull, dirty and dangerous work that is not suitable for human beings to engage in (21). The application of AI can help workers remove themselves from work that they dislike and give them more time to do things that they enjoy (22). It has been demonstrated that the long-term use of AI contributes to increased employment and reduced working hours (23). The destructive power of AI on future employment could be offset by continuous growth in productivity and real incomes (24). In addition, with the creation of a large number of “pleasant jobs” (25), the quality of workers' employment could be much improved, and positive life events that improve the mental health of workers could become more frequent. In addition, technological change promotes the growth of workers' income and further improves the average happiness of workers (26). Although AI is able to perform intuitive and empathetic tasks, it will still take time for AI to replace workers engaged in lower-level tasks, take over some jobs, or even completely supersede human labor (27). This threat of the complete replacement of human labor can be mitigated through appropriate regulatory measures. In a sense, it can also enhance the subjective wellbeing of individuals (28). In conclusion, optimistic scholars believe that AI is more creative than destructive in terms of the economy and society and that its destructive power is controllable. According to these scholars, the widespread use of AI is conducive to increasing the positive life events that improve workers' emotions and effectively improve their mental health.

Currently, AI has not been fully, deeply integrated into the Chinese manufacturing industry (29). Chinese manufacturers have a limited understanding of AI, and they usually use AI to replace certain heavy and mechanical tasks. The methods with which AI is applied are relatively simple (30), and AI integration is not yet sufficiently developed to replace all human work. This means that there has been no large-scale abuse of AI in the manufacturing industry thus far, and AI had a relatively small influence on the negative life events that affect workers' emotions. Moreover, the destructive impact of AI on the mental health of manufacturing workers can be minimized through the provision of lifelong education, increased free time, and the issuance of a universal basic income, which would improve workers' mental health to a certain extent.

In light of this discussion, we propose Hypothesis 1: Compared with workers in manufacturing enterprises that do not use AI, workers in manufacturing enterprises that do adopt AI have significantly improved mental health.

### Mechanism by Which AI Affects the Mental Health of Manufacturing Workers

Regarding the adoption of AI, one view is that enterprises need AI to improve their output efficiency and remain competitive in the market. Therefore, holding circumstances constant, the use of AI improves work efficiency and reduces employee overtime. AI, robots, machine algorithms, etc., can continue to work 24 h a day with a low probability of work accidents and without the need to pay overtime wages, and thus can supplement or even replace the overtime hours of workers. A reduction in overtime hours can help reduce depression and improve mental health. Based on this, we propose Hypothesis 2: The use of AI reduces the probability of overtime work, which in turn benefits workers' mental health.

However, this view has been questioned. Another perspective emphasizes that the appropriate use of AI is to compensate for labor shortages and that the adoption of AI is an alternative measure taken because of the shortage of labor. The greater the labor shortages of enterprises are, the more likely those enterprises are to use AI. The scale of AI adoption is limited. Therefore, in the current Chinese manufacturing industry, the use of AI and overtime work among employees are likely to coexist. In this context, AI is often used first for jobs for which it is difficult to recruit workers and that have a poor working environment. Currently, the scenarios in which AI is applied typically involve dangerous and dirty work environments. Rapid changes to the work environment could have an impact on the occupational safety and health of workers. Existing research has found that AI does play an important role in improving certain aspects of the work environment. In particular, AI increases the flexibility, safety, and convenience of the working environment. Robots are commonly used in harsh, dangerous, inaccessible or unsafe work environments to improve workers' safety as well as their efficiency, productivity and flexibility (31). For example, the application of cutting-edge AI technologies in the mining industry to improve mineral exploration, mine planning, equipment selection, underground and surface equipment operation, drilling and blasting, and mineral processing could help create a safer and more efficient work environment (32). In the construction industry, occupational health and safety management systems can identify hazardous situations and respond autonomously, helping to improve the occupational health and safety of construction workers (33). The application of AI subfields to other aspects of the construction industry has also played a significant role in improving job safety, accuracy, and efficiency (34). In nuclear power plants, AI can reduce the frequency of human error and improve job safety (35). In automated industrial vehicles, the intelligent system controls factors such as speed and distance, thus reducing driver error; as a result, the probability of accidents is extremely low, which ensures the safety of the work environment. In addition, AI is often used to replace labor-intensive, procedural manual routines in static work environments, helping to reduce workloads and increase the ease of work (36, 37). Given the current tendency of Chinese manufacturing enterprises to use AI to replace certain heavy and mechanical tasks, AI mainly helps enhance the flexibility and safety of the work environment and the ease of completing certain tasks. As working environments improve, the mental health and wellbeing of workers are also positively affected. Accordingly, we propose Hypothesis 3: The use of AI improves the work environment, which in turn benefits the mental health of workers.

## Data, Variables and Models

### Data Source and Data Processing

The data used in this paper are derived from the 2018 CLDS, which was organized and implemented by the Social Science Research Center of Sun Yat-sen University. This survey uses a multistage, multilevel and labor-scale probability proportional to size sampling method. The target population is members of the workforce aged 15 to 64 from 29 provinces, municipalities, and autonomous regions nationwide (Hong Kong, Macao, Taiwan, Tibet, and Hainan are excluded). The survey focuses on the status of and changes in individual workers, families and communities. A total of 4,770 individuals, 4,761 families, and 133 communities were involved in the survey, which indicates good representation. In addition, the individual survey in the 2018 CLDS includes a questionnaire specifically focused on information about the individual worker and the use of AI by work units, which is in line with the objectives of this study. The CLDS surveyed 2,547 corporate workers, of whom 1,040, or 33%, were manufacturing workers. Therefore, the empirical data used in this paper come only from the individual questionnaire in the 2018 CLDS.

The sample selection process in this study is as follows: On the basis of our research objectives, we focus on the situations of workers in manufacturing enterprises. The size of the sample of manufacturing workers is 838, including 801 workers under the age of 60. A sample of manufacturing workers aged 16–60 is selected for analysis. Due to a lack of information about unions in the sample, there are 227 observations with missing values. Therefore, the final analysis sample includes 550 valid observations. In addition, the effective sample size used in the robustness tests varies slightly.

### Variable Descriptions

The dependent variable is the mental health status of the manufacturing workers. Depression is an important indicator of mental health status and has often been used in the literature to measure mental health status. In the CLDS questionnaire, respondents were asked to report the frequency with which they experienced 20 different symptoms of depression over the past week. The response options were “never or basically never (<1 day), rarely (1–2 days), often (3–4 days), almost always (5–7 days),” and these responses were assigned values of 0, 1, 2, and 3 points, respectively. A score was calculated for each of the 20 depressive symptoms, and the total score for each respondent was calculated as a proxy indicator for mental health. For this indicator, a higher score implies worse mental health, while a lower score implies that the respondent's mental health is relatively good.

The core independent variable is AI, which originates from a question “Does your employer use technologies such as highly automated processes, robots, or AI (e.g., driverless cars, machine translation, industrial robots, etc.)” in the CLDS questionnaire. If the answer is yes, this variable is assigned a value of 1. If the answer is no, it is assigned a value of 0.

The mediating variables are overtime and the work environment. We use answers to the question “Do you work overtime under normal circumstances?” as the basis for defining the overtime variable. The variable is set to 1 if the answer is yes and set to 0 if it is no. The work environment variable measures workers' evaluations of their satisfaction with their work environment. We use five options, namely, very dissatisfied, not quite satisfied, neither satisfied nor dissatisfied, relatively satisfied, and very satisfied, with the options assigned values of 1, 2, 3, 4, and 5, respectively. The higher the value of this index is, the better the work environment is.

In addition, to prevent other important variables affecting workers' mental health from being missed, in this paper, three categories of control variables are included: individual worker characteristics, employment characteristics, and social capital. First, individual worker characteristics include gender, age, marital status, hukou status, years of education, and physical health, among other variables. Second, employment characteristics include employer type, annual income and labor union status. Third, social capital measures the number of acquaintances and neighborhood support. In addition, given that work-related mental health is affected by regional economic development, regional characteristics are also controlled for. The meanings of each variable and corresponding descriptive statistics are presented in Table 1.

**Table 1:** Variable implications and descriptive statistics.

**Variable name**	**Variable meaning**	**Mean**	**Standard error**	***N***
**Dependent variable**			
Mental health	The total scores of 20 depressive symptoms	7.286	9.043	801
**Core independent variable**			
AI	Unused = 0, used = 1	0.260	0.439	800
**Mediating variable**			
Work overtime	Without overtime = 0, overtime = 1	0.405	0.491	691
Work environment	Work environment satisfaction: very dissatisfied = 1, not quite satisfied = 2, neither satisfied nor dissatisfied = 3, relatively satisfied = 4, very satisfied = 5	3.381	0.787	800
**Controlled variables**			
Gender	Female = 0, male = 1	0.537	0.499	801
Age	Unit: year	39.210	10.090	801
Marital status	Unmarried = 0, married = 1	0.863	0.344	801
Hukou status	Agricultural hukou = 0, non-agricultural hukou = 1	0.313	0.464	800
Years of education	Deprived of education = 0, primary school/private school = 6, middle school = 9, high school (ordinary high school, vocational high school, technical school, technical secondary school) = 12, junior college = 15, undergraduate degree = 16, master or above = 19	10.330	3.319	801
Physical health	Very unhealthy = 1, not quite unhealthy = 2, neither healthy nor unhealthy = 3, relatively healthy = 4, very healthy = 5	3.835	0.817	801
Employer type	State-owned enterprises or collective enterprises = 1, private enterprises (private enterprises, private enterprises, foreign investment, joint ventures, etc.) = 0	0.089	0.284	801
Annual income	Post-tax wage income in 2017, take the logarithm	9.531	3.367	777
Labor union	No union = 0, with the union =1	0.310	0.463	574
The Number of acquaintances	No acquaintance = 1, 1–16 acquaintances = 2, more than 17 acquaintances = 3	1.970	0.509	794
Neighborhood support	Very little = 1, less = 2, generally = 3, more = 4, a great many = 5	3.162	0.985	801
Regional characteristics	Eastern region = 1, Non-Eastern region = 0	0.782	0.413	801

Table 1 shows that the rate of use of AI in the manufacturing industry was 26% in 2018. In other words, AI was used in more than a quarter of all manufacturing companies. This shows that the integration of intelligent technology into the manufacturing industry has begun to be more common, but manufacturing enterprises have blindly adopted AI, and the percentage of firms using AI is still low. The satisfaction of workers with their work environment was relatively high, with an average evaluation score of 3.381, indicating that current work environments are relatively good. The mean and the standard deviation of the mental health variable are 7.286 and 9.043, respectively, indicating that workers have relatively good mental health but that there is wide variation. Male workers account for 53.7% of the sample, and the average age is 39.21 years old. Furthermore, married workers account for 86.3% of the sample, and workers with a nonagricultural hukou account for 31.3%. In addition, the average number of years of education is 10.33, and physical health scores were as high as 3.835. Most of the workers worked in private enterprises, foreign-funded enterprises, joint-venture enterprises, or other enterprises, and only 8.9% worked in state owned or collective enterprises. Most manufacturing enterprises (78.2%) are located in the eastern region. Across the whole sample, 31% of workers were involved in labor unions. Workers have moderate levels of social capital; that is, their number of acquaintances ranges from 1 to 16, and the frequency with which mutual assistance is provided within their neighborhoods is moderate.

### Strategy for Model Setting and Analysis

This paper aims to explore the impact of the trend toward AI adoption in the manufacturing industry on the mental health of workers. And to clarify the role of overtime and the work environment in mediating the impact of AI on mental health. To this end, the empirical model is established as follows:

*Psyhealth*_*i*_ = α_0_+_β_0_*AIi*_+γ_0_*control*_*i*_+ε_*i*_ (Formula 1)

*Overtime*_*i*_ = α_1_+_β_1_*AIi*_+γ_1_*control*_*i*_+ε_*i*_ (Formula 2)

*Psyhealth*_*i*_ = α_2_+_β_2_*AIi*_+δ_1_*Overtime*_*i*_+γ_2_*control*_*i*_+ε_*i*_ (Formula 3)

*Environment*_*i*_ = α_3_+_β_3_*AIi*_+γ_2_*control*_*i*_+ε_*i*_ (Formula 4)

*Psyhealth*_*i*_ = α_4_+_β_4_*AIi*_+δ_2_*Environment*_*i*_+γ_3_*control*_*i*_+ε_*i*_ (Formula 5)

Formula 1 is used to test for the effect of AI on the mental health of manufacturing workers, that is, to examine hypothesis 1. Formula 2 and formula 3 are used to analyze the mediating role of overtime work in the effect of AI on the mental health of manufacturing workers, that is, to examine Hypothesis 2. In addition, formula 4 and formula 5 are used to test whether the work environment mediates the effect of AI on the mental health of manufacturing workers, that is, to test Hypothesis 3. In the formulas, the subscript *i* indicates individual workers and *Psyhealth*_*i*_ indicates worker i's mental health. In addition, *Overtime*_*i*_ represents the overtime practices of worker i. *Environment*_*i*_ indicates worker i's satisfaction with his or her work environment, and *AI*_*i*_ is a dummy variable that indicates whether the enterprise where worker i is employed uses AI technology. Moreover, *control*_*i*_ represents the control variable matrix, which includes variables related to individual characteristics, employment characteristics and social capital. Finally, α_0_, α_1_, α_2_, α_3_ and α_4_ are intercept items, and ε_*i*_ is a random disturbance.

To test the mediation effect, the traditional stepwise method and the popular bootstrap method are employed in this paper. The stepwise method is used to test the significance of the two groups, one group is β_0_, β_1_ and δ_1_, the other group is β_0_, β_3_, δ_2_. If β_0_ is significant, then β_1_ and δ_1_ should be significant, which would suggest that the mediation effect is significant and that overtime work is a partial mediator. If β_0_ is not significant, then β_1_ and δ_1_ should not be significant, which would imply that overtime work is a full mediator. Similarly, the mediation effect of work environment is the same with the derivation process. However, the power of the stepwise method in terms of testing for mediation effects is still controversial. This is why we use the bootstrap method to strengthen our test for mediation effects. N bootstrapped samples are obtained by repeatedly drawing observations from the sample N times, testing the significance of the product of the coefficients, namely, β_1_ δ_1_ and β_3_ δ_2_, in the N bootstrapped samples and splitting the mediation effect into indirect and direct effects. Moreover, to further improve the test's power, the 95% confidence intervals are constructed by using bias correction and acceleration methods. If the confidence intervals do not contain 0, then the results are significant and there is a mediation effect. If the confidence interval for neither the indirect effect nor the direct effect contains 0, the mediation effect is a partial mediation effect. If the confidence interval for the direct effect contains 0, the mediation effect is a full mediation effect.

## Results

### The Overall Effect of AI on the Mental Health of Manufacturing Workers

Model 1 in Table 2 reports the overall effect of AI on the mental health of manufacturing workers. After controlling for individual worker characteristics, employment characteristics and social capital, the regression coefficient for AI is −1.643, a result that is significant at the 5% level. This suggests that the use of AI can effectively reduce the psychological depression scores of manufacturing workers, thereby promoting better mental health among workers. Hypothesis 1 is accepted.

**Table 2:** The impact of AI on the mental health of manufacturing workers: overall and mediating effects.

**Variables**	**Mental health**	**Work overtime**	**Mental health**
	**Model 1**	**Model 2**	**Model 3**
AI	−1.643^**^ (0.758)	0.894^***^ (0.215)	−1.816^**^ (0.774)
Work overtime			0.845 (0.794)
Gender	−1.312^*^ (0.760)	0.359^*^ (0.193)	−1.379^*^ (0.762)
Age	−0.065 (0.044)	−0.027^**^ (0.011)	−0.060 (0.044)
Marital status	−1.760 (1.404)	0.578^*^ (0.320)	−1.866 (1.422)
Hukou status	0.259 (0.888)	−0.341 (0.228)	0.322 (0.892)
Years of education	−0.073 (0.143)	−0.040 (0.033)	−0.066 (0.143)
Physical health	−2.578^***^ (0.496)	−0.140 (0.118)	−2.553^***^ (0.495)
Employer type	0.057 (1.284)	−0.272 (0.348)	0.104 (1.284)
Annual income	−0.036 (0.154)	−0.018 (0.033)	−0.032 (0.156)
Labor union	0.659 (0.878)	0.326 (0.220)	0.600 (0.878)
The number of acquaintances	−1.331^*^ (0.767)	0.197 (0.192)	−1.368^*^ (0.769)
Neighborhood support	−0.011 (0.390)	−0.362^***^ (0.098)	0.057 (0.408)
Regional characteristics	−0.653 (1.021)	0.335 (0.242)	−0.714 (1.026)
Constant	26.524^***^ (4.198)	1.341 (0.848)	25.855^***^ (4.249)
Observations	550	550	550

* *p < 0.1*,

** *p < 0.05*,

*** *p < 0.01*.

In addition, the regression results for Model 1 convey the following information. First, compared with female workers, male workers had significantly lower psychological depression scores by 1.312 points. That is, male workers have relatively good mental health. Second, the positive effect of the physical health of the manufacturing workers on their mental health is relatively large. For each unit of improvement in the physical health of the manufacturing workers, the depression score decreases by 2.578 points. This is much greater than the effect of AI on the mental health of manufacturing workers. Third, the more acquaintances the manufacturing workers have, the lower their psychological depression scores are. That is, the better their mental health is. This suggests that among workers, social capital is conducive to alleviating psychological depression and promoting psychological wellbeing.

### Mediating Effect of Overtime Work on the Mental Health of Manufacturing Workers

Model 1, Model 2, and Model 3 in Table 2 show the step-by-step estimation of the mediating effect of overtime work. The results of Model 1 show that AI can help reduce the psychological depression scores of manufacturing workers and improve their mental health. The results of Model 2 show that there is a positive correlation between AI and overtime. The use of AI does not reduce the likelihood of working overtime; on the contrary, the probability of working overtime. This result lends support to the idea that companies are more likely to adopt AI as a result of difficulties in recruiting. In addition, at this stage, AI is used on a limited scale due to its high input costs. Moreover, the integration of the Chinese manufacturing industry and AI is only just beginning. Therefore, the Chinese manufacturing industry is currently more likely to use AI and require corporate employees to work overtime. The results of Model 3 show that the positive impact of AI on mental health is still significant, while overtime has no significant impact on the mental health of manufacturing workers. This shows that working overtime does not mediate the relationship between AI and the mental health of manufacturing workers. Hypothesis 2 is not supported by the data.

### Mediating Effect of the Work Environment on the Mental Health of Manufacturing Workers

Given the empirical results in Table 2, working overtime has no significant effect on mental health. Therefore, when estimating the mediating effect of the work environment on the mental health of manufacturing workers, overtime work is included as an employment characteristic control variable. In Table 3, Models 4, 5 and 6 present the empirical results of the stepwise test of the mediating effects of the work environment. Model 4 tests the overall effect of AI on the mental health of manufacturing workers. No further elaboration is needed. Model 5 reports the effect of AI on the work environment. Model 6 reports the effect of AI and the work environment on the psychological wellbeing of manufacturing workers. In Model 5, the regression coefficient for the effect of AI on the work environment is significantly positive. This indicates that the use of AI significantly improves the satisfaction of manufacturing workers with their work environment and promotes an improved work environment in the manufacturing industry. In the results of Model 6, the regression coefficients for the effects of AI and the work environment on the psychological health of manufacturing workers are significant. Combined with the significant regression coefficient on AI in Model 4, these results imply that the work environment acts as a mediator between AI and the psychological health of manufacturing workers. Hypothesis 3 is confirmed. The trend of intelligent technology use in the manufacturing industry improves the psychological health of manufacturing workers by promoting an improved work environment.

**Table 3:** The impact of AI on the mental health of manufacturing workers: overall and mediating effects.

**Variables**	**Mental health**	**Work environment**	**Mental health**
	**Model 4**	**Model 5**	**Model 6**
AI	−1.816^**^ (0.774)	0.157* (0.080)	−1.607^**^ (0.765)
Work environment			−1.332^***^ (0.509)
Gender	−1.379^*^ (0.762)	−0.162^**^ (0.067)	−1.595^**^ (0.772)
Age	−0.060 (0.044)	0.002 (0.004)	−0.057 (0.043)
Marital Status	−1.866 (1.422)	0.063 (0.111)	−1.782 (1.399)
Hukou status	0.322 (0.892)	0.180^**^ (0.083)	0.561 (0.892)
Years of education	−0.066 (0.143)	0.014 (0.012)	−0.048 (0.141)
Physical health	−2.553^***^ (0.495)	0.176^***^ (0.046)	−2.318^***^ (0.472)
Employer type	0.104 (1.284)	−0.374^***^ (0.124)	−0.394 (1.298)
Annual income	−0.032 (0.156)	−0.015 (0.012)	−0.052 (0.153)
Labor union	0.600 (0.878)	0.040 (0.079)	0.652 (0.879)
Work overtime	0.845 (0.794)	−0.118 (0.072)	0.688 (0.790)
The number of acquaintances	−1.368^*^ (0.769)	0.040 (0.071)	−1.315^*^ (0.762)
Neighborhood support	0.057 (0.408)	−0.016 (0.035)	0.036 (0.405)
Regional characteristics	−0.714 (1.026)	0.086 (0.097)	−0.600 (1.005)
Constant	25.855^***^ (4.249)	2.530^***^ (0.324)	29.224^***^ (4.739)
Observations	550	550	550

* *p < 0.1*,

** *p < 0.05*,

*** *p < 0.01*.

To enhance the power of the stepwise analysis, the bootstrap method is also employed in this paper to test for mediation effects. A total of 1,000 manufacturing worker samples were drawn, and the results tested by using the bias-corrected and accelerated (Bca) 95% confidence intervals.

The results show that the Bca 95% confidence interval for overtime work includes 0, indicating that overtime is not a mediator. The Bca 95% confidence interval for the work environment does not contain 0: the mediating effect of the work environment remains significant (the confidence intervals for neither the indirect nor the direct effect contained 0). The work environment explains 11.509% of the psychological wellbeing of manufacturing workers.

### Robustness Test

To ensure the reliability of the above findings, two robustness tests are conducted (see Table 4 for the test results). The previous empirical results show that overtime work does not exhibit any mediating effects. Therefore, in this section, only the robustness of the main effect of AI and the mediating effect of the work environment are tested.

**Table 4:** The impact of AI on the mental health of manufacturing workers: a robustness tests.

**Variables**	**Psychological depression**	**Overall work environment**	**Mental health**
	**Model 7**	**Model 8**	**Model 9**
AI	−0.531^*^ (0.282)	0.536^*^ (0.278)	−1.527^**^ (0.766)
Work overtime		−1.033^***^ (0.2)	0.381 (0.818)
Overall work environment			−0.442^***^ (0.142)
Gender	−0.261 (0.232)	−0.411^*^ (0.239)	−1.605^**^ (0.761)
Age	−0.016 (0.014)	0.010 (0.015)	−0.056 (0.043)
Marital status	−0.540 (0.355)	0.417 (0.403)	−1.704 (1.399)
Hukou status	0.193 (0.273)	0.476 (0.306)	0.443 (0.889)
Years of education	−0.016 (0.040)	0.033 (0.043)	−0.052 (0.139)
Physical health	−0.466^***^ (0.142)	0.833^***^ (0.158)	−2.173^***^ (0.475)
Employer type	−0.348 (0.442)	−0.815^*^ (0.449)	−0.193 (1.278)
Annual income	−0.007 (0.038)	−0.039 (0.049)	−0.050 (0.151)
Labor union	0.206 (0.270)	0.256 (0.274)	0.733 (0.881)
The number of acquaintances	−0.491^**^ (0.241)	0.193 (0.251)	−1.210 (0.758)
Neighborhood support	0.137 (0.119)	0.073 (0.133)	0.073 (0.398)
Regional characteristics	0.063 (0.288)	0.504 (0.319)	−0.525 (1.005)
Constant	2.286^**^ (1.022)	12.111^***^ (1.180)	31.139^***^ (4.959)
Observations	547	547	547

* *p < 0.1*,

** *p < 0.05*,

*** *p < 0.01*.

First, the robustness of the overall effect is tested by using an alternative measurement for the dependent variable. In the previous section, workers' psychological wellbeing was measured as the sum of the scores for 20 depressive symptoms. Here, drawing on the existing literature, a cutoff of 16 points is used to construct an indicator for worker tendencies toward psychological depression as a proxy variable for mental health. If a worker's total depressive symptom score is strictly <16, then his or her mental health status is considered good, and a value of 0 is assigned to the variable. Conversely, if the total score is 16 or greater, it indicates poor mental health, and a value of 1 is assigned. The regression results are obtained by substituting this new variable into formula 1 and are presented as Model 7. The results are highly consistent with those of Model 1, with the regression coefficient for AI remaining negative and significant. This suggests that AI does help reduce manufacturing workers' tendency toward psychological depression and enhances workers' mental health.

Second, the robustness of the mediating effect was tested by using a new measurement for the mediating variable. Whereas in the previous section, only a single-dimensional measure of the work environment was used, here, a five-dimensional measure is used. The five dimensions include job income, job security, work environment, working hours and overall job satisfaction. The sum of the satisfaction scores for the five dimensions is used as a proxy for the work environment. The larger the indicator is, the better the overall work environment. The stepwise approach (Model 7 to Model 8) and bootstrap method are used to test for the mediating effects of this indicator (third row of Table 5). The results show that the effects of AI and of the overall work environment on the mental health of manufacturing workers are still significant, and the Bca 95% confidence interval does not contain 0. The work environment is a robust mediator of the relationship between AI and manufacturing workers' psychological wellbeing.

**Table 5:** The impact of work environment on the psychological well-being of manufacturing workers: a test of mediating effects based on the Bootstrap method.

**Mediators**		**Coeff**	**Boot SE**	**95% CI(Bca)**	**Change, %**
Work overtime	Indirect effect	0.173	0.175	[−0.104, 0.592]	—
	Direct effect	−1.816	0.810	[−3.389, −0.207]	
Work environment	Indirect effect	−0.209	0.136	[−0.642, −0.016]	11.509
	Direct effect	−1.607	0.805	[−3.202, −0.039]	
Overall work environment	Indirect effect	−0.237	0.150	[−0.604, −0.014]	13.435
	Direct effect	−1.527	0.763	[−3.178, −0.038]	

## Further Discussion: Heterogeneity Analysis

The impact of AI on workers' mental health has been found to vary with differences in workers' endowments. For this reason, this study continues to explore the heterogeneity in the effects of AI on the mental health of manufacturing workers along two dimensions: skill levels and generational membership. These two dimensions are chosen for the following reasons. First, at the beginning of the intelligentization of manufacturing, AI is usually used to replace routine, simple, and low-skilled work (38). It is usually low-skilled workers who are employed in this type of work. This means that low-skilled workers are exposed to AI earlier than high-skilled workers. Moreover, with the deepening of the integration of AI into manufacturing, manufacturing enterprises increasingly need workers with advanced digital skills but have a reduced demand for low-skilled workers (39). Therefore, the impact of AI in manufacturing on workers' mental health is also expected to vary by skill level. Second, unlike workers born before 1980, workers born after 1980 were born after the reform and opening up, and they are relatively better able to learn and accept new technologies such as AI. Therefore, should we also expect there to be generational differences in the impact of AI on the mental health of manufacturing workers?

### Heterogeneity in the Impact of AI on the Mental Health of Manufacturing Workers With Different Skill Levels

In this paper, workers are categorized according to their level of education into those with a middle school education or lower and those with a high school education or higher as a way of distinguishing between low-skilled and high-skilled manufacturing workers. Table 6 reports the heterogeneity in the impact of AI on the mental health of manufacturing workers with different skill levels. The results show that the impact of AI on the mental health of manufacturing workers varies significantly with skill levels. Specifically, AI significantly improves the mental health of low-skilled manufacturing workers but not that of high-skilled manufacturing workers. Notably, the coefficient for the effect of AI on the mental health of low-skilled manufacturing workers is 2.342, a positive effect much higher than that estimated for the full sample, and this result is significant at the 5% level.

**Table 6:** Heterogeneity in the impact of AI on the mental health of manufacturing workers: low-skilled versus high-skilled.

**Variables**	**Low-skilled**	**High-skilled**
	**Model 10**	**Model 11**
AI	−2.342^**^ (1.036)	−0.873 (1.226)
Controlled variable	Controlled	Controlled
Constant	24.775^***^ (5.767)	29.458^***^ (7.932)
Observations	308	242

* *p < 0.1*,

** *p < 0.05*,

*** *p < 0.01*.

*The control variables are the same as in Model 1. The focus is on skill heterogeneity in the impact of AI on the mental health of manufacturing workers. Regression results for control variables are not presented in detail for brevity*.

There are several possible reasons for this finding. First, while AI replaces specific tasks completed by low-skilled workers that are often dull, dangerous, dirty, etc., this does not mean that low-skilled workers are willing to do such jobs. On the contrary, the use of AI frees low-skilled workers from these jobs, giving them time to do what they are willing to do and promoting their mental health. Second, as high-skilled workers are usually engaged in more knowledge-based work, AI acts as more of a support for them. This means that AI is relatively less helpful to high-skilled workers, and thus, the mental health of high-skilled workers does not fluctuate significantly. Third, as AI in China is currently mainly used for dull, dangerous, and dirty tasks and other tasks usually completed by low-skilled workers, there is thus a greater gain in mental health among low-skilled workers than among manufacturing workers as a whole.

### Heterogeneity in the Impact of AI on the Mental Health of Manufacturing Workers Across Generations

In this paper, 1980 is used as the generational cutoff for manufacturing workers, with those born in 1979 or before being referred to as pre-1980's workers and those born in 1980 or after being referred to as post-1980's workers. Table 7 reports the heterogeneity in the impact of AI on the mental health of manufacturing workers across different generations. The results show that there is a significant generational effect. Specifically, when AI is used in the manufacturing industry, the mental health scores of the pre-1980's manufacturing workers increases by 2.070 points, a result that is significant at the 10% level. However, there is no clear positive effect of AI on the psychological wellbeing of post-1980's manufacturing workers. This suggests that AI helped to promote the mental health of only the pre-1980's manufacturing workers.

**Table 7:** Heterogeneity in the impact of AI on the mental health of manufacturing workers: pre-1980's vs. post-1980's.

**Variables**	**pre-1980's**	**post-1980's**
	**Model 12**	**Model 13**
AI	−2.070^*^ (1.079)	−1.414 (1.076)
Controlled variable	Controlled	Controlled
Constant	14.019^**^ (5.873)	30.419^***^ (7.385)
Observations	287	263

* *p < 0.1*,

** *p < 0.05*,

*** *p < 0.01*.

*The control variables are the same as in Model 1. The focus is on skill heterogeneity in the impact of AI on the mental health of manufacturing workers. Regression results for control variables are not presented in detail for brevity*.

There are two possible reasons for this result. First, compared with the post-1980's workers, the pre-1980's workers are harder working and more willing to do dirty, hard and tiring work to earn an income. When AI takes on such work, the workloads of the pre-1980's workers are eased, and their mental health is improved. Second, unlike the post-1980's workers, the pre-1980's workers are past their career peak and are about to exit the labor market. For these workers, work is not their only priority, and family happiness becomes their main pursuit. The use of AI increases the flexibility in their working hours, allowing them to spend more time with their families and receive greater moral support from them, thus improving their mental health.

## Conclusions and Suggestions

### Conclusions

Using data from the 2018 CLDS, we examine the impact of AI and the mediating effect of the overtime and work environment on the mental health of manufacturing workers. We further analyze the heterogeneity in the effects of AI on the mental health of manufacturing workers by skill level and generation. In conclusion, AI has an obvious positive effect on the mental health of manufacturing workers. There is a positive relationship between AI and overtime work but no mediating effect between overtime work and manufacturing workers' mental health. This study also confirms that AI improves the work environments of manufacturing workers and thus indirectly promotes their mental health. The quality of the working environment mediates the impact of AI on the mental health of manufacturing workers. This study still emphasizes that the contribution of AI to the mental health of manufacturing workers varies by skill level and generation. AI can contribute significantly to improvements in the mental health of low-skilled manufacturing workers and those workers born before 1980 and has a greater positive impact on low-skilled workers.

This study had three main limitations. First, this study is limited by the use of cross-sectional data. Although the CLDS is data longitudinal survey, only the 2018 wave contains information that applies to this research topic, as the AI questions were first asked in 2018. The cross-sectional nature of the data makes it difficult to conduct an in-depth examination of the mechanisms underlying the impact of the working environment on the mental health of manufacturing workers. We will continue to focus on related AI data in the future in an effort to develop a panel dataset to subsequently advance such research. Second, this study is constrained by the available data measurements. In reality, the path by which AI impacts the mental health of manufacturing workers is highly complex. AI does not affect mental health through only one pathway, such as the working environment, which was studied in this paper. AI may affect the mental health of manufacturing workers through opportunities for promotion, social status, and work–family balance, among other pathways. More data and information are still needed to answer this question. Third, this study is limited by the survey structure. Since the unemployed do not answer the question “Does your employer use technologies such as highly automated processes, robots, or AI (e.g., driverless cars, machine translation, industrial robots, etc.)?,” it is difficult to estimate the mental health status of those who have lost their jobs due to AI. It is only possible to observe the impact of AI on the mental health of those currently working in manufacturing.

This study contributes a micro perspective for understanding the development dividends received by workers as AI and manufacturing become deeply integrated. Through this integration, people have a greater sense of gain from sharing in the achievements of intelligent manufacturing, which is reflected in their subjective psychological wellbeing. The impact of AI on the workforce is not only reflected in objective measures of economic wellbeing, such as the distribution of employment income but should also ultimately affect the subjective psychological wellbeing of workers. The key to this process is the improvement of the work environment through the use of AI, which in turn enhances the mental health of manufacturing workers. Currently, intelligent technology in China's manufacturing industry has mainly replaced work in unfavorable environments. Low-skilled workers and workers born before 1980 are more likely to work in poor work environments, such as those of dull, dirty and dangerous jobs. Therefore, in the initial stages of the development of intelligent technology in the Chinese manufacturing industry, the mental health of workers is significantly higher, and low-skilled and pre-1980's workers derive greater mental health benefits from AI. Relatively speaking, highly skilled workers and workers born after 1980 have yet to enjoy the psychological benefits of AI. The impact of AI on the mental wellbeing of manufacturing workers exhibits poverty spillover effects.

### Suggestions

With the trend toward the deep integration of AI and manufacturing, the question of how to protect workers' mental health, reduce psychological injuries, and encourage workers to share in the dividends of AI development is still worth considering. This paper makes the following two suggestions.

First, AI should be continually utilized to improve the work environments within the manufacturing industry in order to enhance the mental health and wellbeing of workers. The positive role of AI in improving the manufacturing work environment should be amplified. Work that workers are unable or unwilling to perform and that is conducted in unfavorable working environments should be handed over to AI to alleviate negative emotions about work and improve workers' mental health. Currently, manufacturing companies, constrained by both investment capital and industrial data, mainly use AI to replace dull, dirty and dangerous jobs in order to cope with the cost pressures arising from recruitment difficulties and expensive labor. However, it is important to note that the fundamental motivation behind the adoption of AI is to improve productivity through technological adjustments and that reducing labor costs is only a secondary consideration. As manufacturing enterprises become better able to control AI, these two constraints will gradually be relaxed. Manufacturers will eventually apply AI to other areas to improve production efficiency, and workplace improvements will no longer be a priority. As a result, companies should use AI cautiously in order to sustain its positive role in improving the work environment, and they should use AI to perform repetitive, fatiguing and dangerous tasks in order to reduce the negative impacts of AI expanding into other areas, which could endanger workers' mental health.

Second, it should be noted that there is a need to develop and design new AI to improve the mental health of highly skilled, post-1980's workers and to expand the benefits of AI for the psychological wellbeing of the workforce as a whole. This paper found no significant impact of AI on the mental health of highly skilled workers or those born after 1980. This is not promising. The number of highly skilled workers is gradually increasing as the average number of years of education increases. In addition, generational turnover in the workforce has made post-1980's workers the largest share of the labor force. This means that AI does not have a clearly positive impact on the mental health of the future workforce. Thus, it is necessary to focus on stimulating the positive impact of AI on the mental health of highly skilled and post-1980's workers. AI must not be used only to complete dull, dirty and dangerous jobs but also to support highly skilled and post-1980's workers. Taking into account their psychological needs, new AI should be developed and designed to reduce their workloads and contribute to their physical and mental wellbeing, thereby expanding the psychological benefits of AI to the entire workforce.

## Data Availability Statement

The data analyzed in this study is subject to the following licenses/restrictions: The 2018 CLDS data needs to wait until it is disclosed to all academic colleagues, and apply for real-name registration on the website of the Center for Social Science Survey at Sun Yat-sen University in Guangzhou, China. After approval, it can be downloaded and used for free. Requests to access these datasets should be directed to https://sysu.pagaloo.com/d/edu.cn/css.

## Author Contributions

LL has sorted out relevant literature on the impact of artificial intelligence and work environment on workers' mental health, designed empirical models and analysis strategies, and participated in data cleaning, empirical results interpretation, and draft writing. WW designed the study, provided instructive comments unremittingly, and participated in manuscript revisions. Both authors contributed to the article and approved the submitted version.

## Funding

This study was supported by grants from the High-level Innovation team and the Outstanding Scholars Program of Guangxi Colleges and Universities. As well as the New urbanization and public policy research and innovation team headed by WW of Guangxi University.

## Conflict of Interest

The authors declare that the research was conducted in the absence of any commercial or financial relationships that could be construed as a potential conflict of interest.

## Publisher's Note

All claims expressed in this article are solely those of the authors and do not necessarily represent those of their affiliated organizations, or those of the publisher, the editors and the reviewers. Any product that may be evaluated in this article, or claim that may be made by its manufacturer, is not guaranteed or endorsed by the publisher.
